# A Simple Three-Step Method for Design and Affinity Testing of New Antisense Peptides: An Example of Erythropoietin

**DOI:** 10.3390/ijms15069209

**Published:** 2014-05-26

**Authors:** Nikola Štambuk, Zoran Manojlović, Petra Turčić, Roko Martinić, Paško Konjevoda, Tin Weitner, Piotr Wardega, Mario Gabričević

**Affiliations:** 1 Center for Nuclear Magnetic Resonance, Ruđer Bošković Institute, Bijenička cesta 54, 10002 Zagreb, Croatia; E-Mail: pkonjev@irb.hr; 2 Croatian Institute for Toxicology and Antidoping, Borongajska 83 g, 10000 Zagreb, Croatia; E-Mail: zoran.manojlovic@antidoping-hzta.hr; 3 Department of Pharmacology, Faculty of Pharmacy and Biochemistry, University of Zagreb, Domagojeva 2, 10000 Zagreb, Croatia; E-Mail: pturcic@pharma.hr; 4 Department for Clinical Pathophysiology, Clinical Hospital Centre Split, Šoltanska 1, 21000 Split, Croatia; E-Mail: roko.romast@gmail.com; 5 Department of General and Inorganic Chemistry, Faculty of Pharmacy and Biochemistry, University of Zagreb, Ante Kovačića 1, 10000 Zagreb, Croatia; E-Mails: tweitner@pharma.hr (T.W.); mariog@pharma.hr (M.G.); 6 NanoTemper Technologies GmbH, Flößergasse 4, 81369 Munich, Germany; E-Mail: piotr.wardega@nanotemper.de

**Keywords:** erythropoietin, antisense, peptide, binding, fluorescence, spectroscopy, thermophoresis, modeling

## Abstract

Antisense peptide technology is a valuable tool for deriving new biologically active molecules and performing peptide–receptor modulation. It is based on the fact that peptides specified by the complementary (antisense) nucleotide sequences often bind to each other with a higher specificity and efficacy. We tested the validity of this concept on the example of human erythropoietin, a well-characterized and pharmacologically relevant hematopoietic growth factor. The purpose of the work was to present and test simple and efficient three-step procedure for the design of an antisense peptide targeting receptor-binding site of human erythropoietin. Firstly, we selected the carboxyl-terminal receptor binding region of the molecule (epitope) as a template for the antisense peptide modeling; Secondly, we designed an antisense peptide using mRNA transcription of the epitope sequence in the 3'→5' direction and computational screening of potential paratope structures with BLAST; Thirdly, we evaluated sense–antisense (epitope–paratope) peptide binding and affinity by means of fluorescence spectroscopy and microscale thermophoresis. Both methods showed similar *K*_d_ values of 850 and 816 µM, respectively. The advantages of the methods were: fast screening with a small quantity of the sample needed, and measurements done within the range of physicochemical parameters resembling physiological conditions. Antisense peptides targeting specific erythropoietin region(s) could be used for the development of new immunochemical methods. Selected antisense peptides with optimal affinity are potential lead compounds for the development of novel diagnostic substances, biopharmaceuticals and vaccines.

## 1. Introduction

In the last two decades antisense peptides became a valuable tool for deriving new biologically active molecules and performing peptide–receptor modulation [1,2,3,4,5,6,7]. They have been used in biomedicine for efficient modeling of more than 40 peptide–receptor systems [1,2,3,4,5,6,7].

This study presents a simple and efficient three-step procedure for the antisense peptide design and the verification of its binding. The first step is the selection of molecular target, *i.e.*, the selection of the targeted epitope, which serves as a starting point for the design of an antisense peptide ligand (paratope). The second step is the rational design of the antisense peptide paratope directed against the selected epitope. Finally, the third step is the evaluation of sense–antisense peptide (epitope–paratope) binding by means of fluorescence spectroscopy and microscale thermophoresis, or other appropriate physicochemical, immunologic or chromatographic technique.

**Figure 1:**
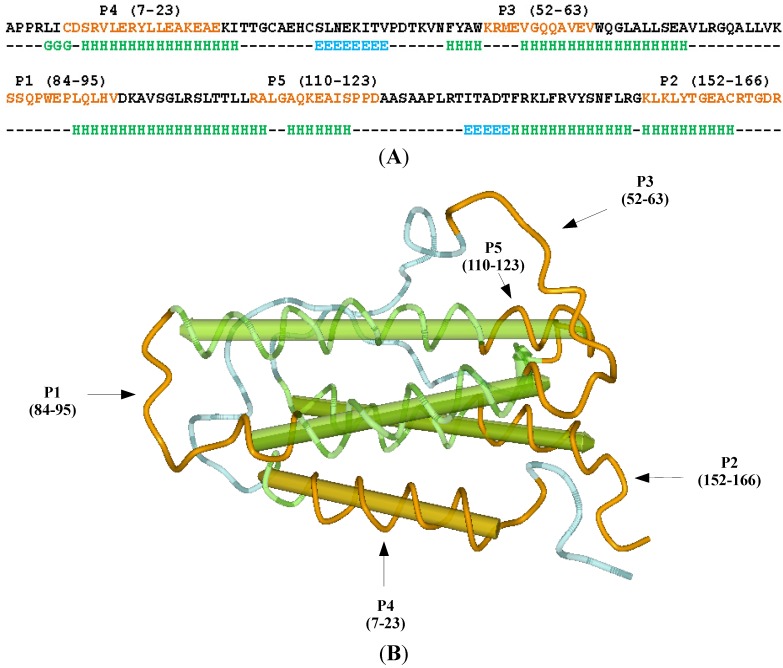
(**A**) Primary and secondary structure of the erythropoietin molecule. G, 3_10_-helix (light green); H, α-helix (light green); E, β-strand (light blue); –, coil/other. P1–P5 are epitope regions of the molecule (light brown) [11]; (**B**) Tertiary structure of the erythropoietin with its bioactive regions P1–P5 (light brown).

Erythropoietin (EPO) is the primary humoral regulator of red blood cells production, *i.e.*, erythropoiesis. It is a large glycoprotein with 193 amino acid residues (signal peptide 1–27, chain 28–193), and molecular weight of approximately 35,000 (Figure 1) [8,9,10,11]. In medicine, EPO is used for therapeutic and diagnostic purposes [8,9,10]. The main therapeutic use is the treatment of anemia resulting from chronic kidney disease, while the measurement of serum levels of EPO is important in differentiating primary polycythemia from secondary polycythemia [8,9]. EPO derivatives also have a history of use as doping agents, especially in endurance sports, because they enhance delivery of oxygen to the tissues [10].

Due to its pharmacologic relevance erythropoietin molecule is well characterized and suitable for the investigation of potential binding sites, which may be of importance for diagnostic and therapeutic procedures involving EPO and its derivatives [8,9,10]. Consequently, we chose EPO as a starting point for the design of specific ligand based on antisense peptide technology.

## 2. Results and Discussion

### 2.1. Selection of the Molecular Target (Epitope): Step 1

The first step in antisense peptide design is the selection of the molecular target, *i.e.*, appropriate epitope. The principal immunogenic regions/epitopes in vaccine design and related immunochemical procedures are interaction sites characterized by high values of hydrophilicity, surface probability and antigenic index [12,13,14]. An alternative approach to the detection of protein interaction sites is the Resonant Recognition Method (RRM), based on the Fourier signal analysis of the amino acid electron–ion interaction pseudo-potential (EIIP) [15,16].

**Figure 2:**
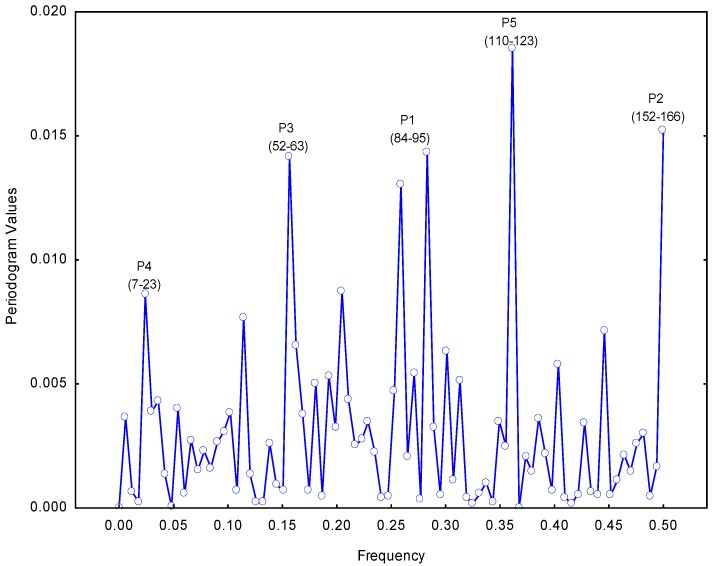
Single spectrum (Fourier) analysis of the erythropoietin after assigning electron–ion interaction pseudo-potential (EIIP) to each amino acid. Frequency = number of cycles per observation; Periodogram Value = (sine coefficient_k_^2^ + cosine coefficient_k_^2^) ×*N*/2; *N* = number of observations in the series. Five dominant frequency peaks identified by the periodogram correspond to the immunogenic parts P1–P5 of the erythropoietin molecule.

The comparison of these two different methods for the prediction of antibody–protein interaction sites is given in Table 1 [13,15]. Classic Hopp and Woods hydrophilicity scale measures averaged sums of solvent parameter values in kcal/mol within the fixed length of the window (e.g., 6 aa) to extract the information on the most likely antigenic parts of the molecule [13,14]. Resonant Recognition Method is a physical and mathematical procedure that interprets protein sequence linear information of the electron–ion interaction pseudo-potential in Rydberg’s unit of energy (Ry), using signal analysis in order to extract regions relevant for intermolecular interactions [15]. The correlation between Hopp and Woods and EIIP values of the individual amino acids in Table 1 is negligible (*r* = 0.162, *p* > 0.05), and similar is valid for the EPO sequence comparison in Figure 1 (*r* = 0.323, *p* < 0.05), a fact suggesting different information qualities of both methods [17]. When both methods are applied to EPO molecule the same peaks, *i.e.*, five hot spots P1–P5, are extracted (Figure 2). This points to high information content of the selected sites.

**Table 1:** Values of amino acid hydrophilicity and electron–ion interaction pseudo-potential (EIIP) [13,15].

Amino Acid	Abbreviation	Hopp and Woods Hydrophilicity	EIIP (Ry)
Arginine	R	3.0	0.0959
Lysine	K	3.0	0.0371
Aspartic acid	D	3.0	0.1263
Glutamic acid	E	3.0	0.0058
Serine	S	0.3	0.0829
Asparagine	N	0.2	0.0036
Glutamine	Q	0.2	0.0761
Proline	P	0.0	0.0198
Glycine	G	0.0	0.0050
Threonine	T	−0.4	0.0941
Histidine	H	−0.5	0.0242
Alanine	A	−0.5	0.0373
Cysteine	C	−1.0	0.0829
Methionine	M	−1.3	0.0823
Valine	V	−1.5	0.0057
Leucine	L	−1.8	0.0000
Isoleucine	I	−1.8	0.0000
Tyrosine	Y	−2.3	0.0516
Phenylalanine	F	−2.5	0.0946
Tryptophan	W	−3.4	0.0548

Specific antibody binding has been experimentally verified by Fibi *et al.* for the regions P2, P4 and P5, respectively (Figure 1) [11]. Out of those regions carboxyl-terminal domain P2 was found to be involved in the biologic function of recombinant human EPO and its receptor binding site. This was verified *in vitro* using cell proliferation assay based on specific inhibition of the EPO activity with P2 induced antisera [11]. Considering the consistence of the models with experimental data (Figure 2), as well as its functional importance, we selected P2 domain as a target epitope for the next procedure steps, *i.e.*, the antisense peptide design (Step 2) and the verification of its binding affinity (Step 3).

### 2.2. Modeling of an Ansisense Peptide (Paratope): Step 2

**Figure 3:**
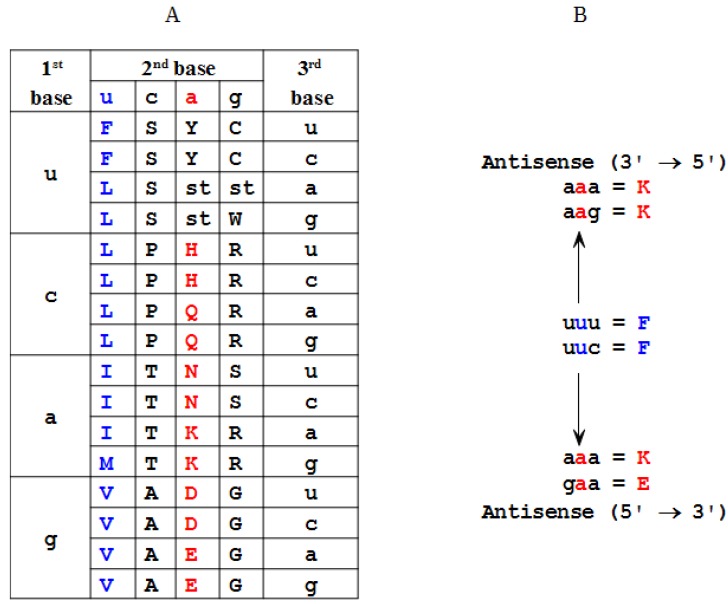
Genetic code table defines 64 nucleotide base triplets of the mRNA, that code for 20 amino acids and 3 stop codons. (**A**) Second nucleotide base of the codon triplet codes the majority of nonpolar (blue) and polar (red) amino acids; (**B**) Antisense peptides based on mRNA transcription in the 3'→5' direction have less antisense amino acids then those transcribed in the 5'→3' direction. u = U, uracil; a = A, adenine; c = C, cytosine; g = G, guanine.

Antisense peptide design is closely related to the genetic code structure [1,2,3,4,5,6]. Sixty-four codons of the genetic code consist of three nucleotide bases. Sixty-one codons are for 20 amino acids and three are stop signals [1]. The antisense sequences are obtained from the mRNA by transcribing uracil (U) into its complement adenine (A) and cytosine (C) into its complement guanine (G), or *vice versa* (Figure 3) [1]. During the last two decades growing experimental evidence supported the thesis that sense and antisense mRNAs define peptides that interact with increased probability [1,2,3,4,5,6]. This biologic phenomenon is closely related to the Proteomic Code, a set of rules by which the information contained in DNA/RNA sequences is transferred to the physicochemical characteristics of the amino acids, protein structure and specific protein–protein interactions [1,2,18]. The concept of antisense peptide based modeling of ligand–receptor interactions has been successfully applied to many receptor systems, and it became a valuable procedure for the design of new bioactive peptides and antibodies [1,2,3,4,5,6].

Biro, Mekler and Idlis first discussed genetic coding of possibly interacting, specific complementary (antisense) amino acids [2,18]. Root-Bernstein, Blalock, Siemion and others investigated the applications of complementary (antisense) peptides, and critically examined the relevance of such a molecular recognition for the modeling of protein/peptide interactions in biomedicine [1,2,3,4,5,6,7,18,19,20,21,22,23,24]. Two main characteristics of the antisense peptide modeling based on the standard genetic code are: (1) tendency for opposite polarity patterns of an antisense peptide when compared to the sense peptide structure; (2) different number of antisense peptides depending on the direction of the mRNA transcription (from left to right and *vice versa*; 3'→5'/5'→3') [1,4,6,24].

Frequently observed molecular interaction of sense and antisense peptide pairs is related to the genetic code property that codons for hydrophobic amino acids are in most cases complemented with the hydrophilic ones, while the neutral ones are complemented mutually [1,2,3,4,6,20]. This fact is illustrated in Figure 3, and results from the triplet codon based structure-function relationship. The second (central) nucleotide base of the codon triplet specifies the majority of nonpolar and polar amino acids [3]. Consequently, most of the codons containing central uracil and adenine code for the amino acids of opposite polarity, and the polarity pattern of antisense peptides is reversed (Figure 3).

The transcription of the sense peptide into the antisense one may be done in the 3'→5' and/or 5'→3' direction. However, from the standpoint of the efficient modeling it is more convenient to use the 3'→5' direction as a template for an antisense peptide design, since, as presented in Figure 3 and Table 2, it results in a significantly fewer peptide structures [4,6,24]. When triplet codons encoding sense amino acids are transcribed in the 3'→5' direction there are 27 possible antisense pairs for 20 amino acids, and when they are transcribed in the 5'→3' direction there are significantly more possibilities, *i.e.*, 52 antisense for 20 sense amino acids (Figure 3, Table 2). This results from the fact that the transcription of antisense mRNA in 5'→3' direction specifies triplet codons of the genetic code table backwards (3rd2nd1st base instead of 1st2nd3rd base, Figure 3).

**Table 2:** Significantly more antisense amino acids are obtained by the mRNA codon transcription in the 5'→3' direction then in the 3'→5' direction.

Amino Acid	Antisense 3'→5'	Antisense 5'→3'
F	K	K, E (η)
L	D, E, N (α)	E, Q, K (θ)
I	Y	N, D, Y (ι)
M	Y	H
V	H, Q (β)	H, D, N, Y (κ)
S	S, R (γ)	G, R, T, A (λ)
P	G	G, W, R (μ)
T	W, C (δ)	G, S, C, R (ν)
A	R	R, G, S, C (ξ)
Y	M, I (ε)	I, V (ο)
H	V	V, M (π)
Q	V	L
N	L	I, V (ρ)
K	F	F, L (ς)
D	L	I, V (σ)
E	L	L, F (τ)
C	T	T, A (υ)
W	T	P
R	A, S (ζ)	A, S, P, T (φ)
G	P	P, S, T, A (χ)

Antisense peptide technology gave promising results in neuroendocrine and immune research, especially with the respect to antibody and paratope design [1,2,3,4,5,6]. Therefore, we applied this theoretical concept to design an antisense peptide targeting receptor-binding site of the human erythropoietin. Carboxyl-terminal domain peptide LKLYTGEACRTGDR, *i.e.*, EPO-P2 epitope (152–166 aa), was used as a template for the antisense peptide design.

The algorithm of antisense mRNA transcription in the 3'→5' direction was applied to generate all possible antisense peptides representing potential paratopes that may bind targeted EPO-P2 region (Table 2 and Table 3) [6,24]. Potential antibody structures (paratopes) to EPO-P2 epitope were selected using Basic Local Alignment Search Tool (BLAST), with blastp option (protein–protein BLAST) [24,25]. The results presented in Table 3 show three antisense pentapeptide motifs contained in human antibody structures (αFαεδ, PLRTζ and ζδPLζ—where α = (D, E, N); δ = (W, C); ε = (M, I); ζ = (A, S)).

**Figure 4:**
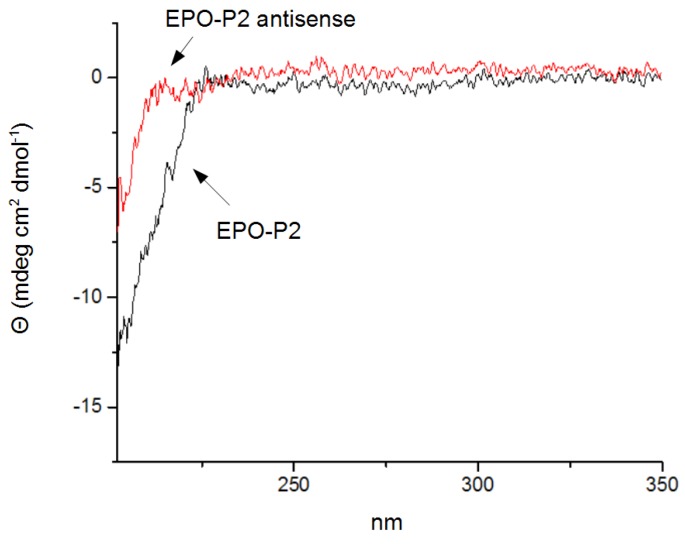
Circular dichroism spectra of Erythropoietin (EPO)-P2 peptide and its antisense.

The final structure of the antisense peptide DFDIWPLRTAWPLS, presented in Table 4, was obtained by joining three linear paratope motifs DFDIW, PLRTA and WPLS, selected on the basis of the highest score of antibody homologies detected by BLAST search. Following this step, EPO-P2 epitope LKLYTGEACRTGDR and its antisense peptide DFDIWPLRTAWPLS were synthesized. The structures of both peptides were analyzed with CD spectroscopy (Figure 4). A strong negative peak with maximum around 200 nm was typical for random coil structure, and the absence of any peaks at other wavelengths indicated no organized structure in both peptides.

**Table 3:** *In silico* paratope scan of erythropoietin P2 epitope (152–166) using Basic Local Alignment Search Tool (BLAST) [24,25].

Antisense Paratope	EPO-P2 Epitope KLFLYTGEACRTGDR	Number of Paratopes	BLAST Detected Antibodies
1	FαFαε	18	6
2	αFαεδ	36	20
3	FαεδP	12	0
4	αεδPL	12	0
5	εδPLR	4	0
6	δPLRT	2	0
7	PLRTζ	4	2
8	LRTζδ	4	0
9	RTζδP	4	4
10	TζδPL	4	6
11	ζδPLζ	8	12

α = (D, E, N); δ = (W, C); ε = (M, I); ζ = (A, S).

**Table 4:** Antisense peptide DFDIWPLRTAWPLS obtained by joining three linear paratope motifs, starting from the antisense paratope 2 selected on the basis of highest number of antibody homologies detected by BLAST.

Antisense Paratope 2	Antisense Paratope 7	Antisense Paratope 11	BLAST Detected Antibodies
DFDIW	PLRTA	WPLS	17

### 2.3. Evaluation of Peptide Binding: Step 3

**Figure 5:**
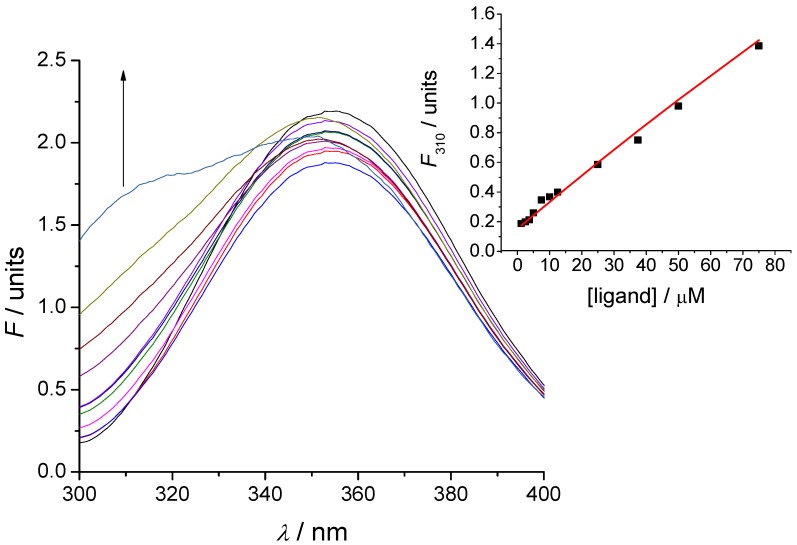
A titration of 2.5 μM solution of EPO-P2 antisense peptide DFDIWPLRTAWPLS with EPO-P2 epitope LKLYTGEACRTGDR, at 25 °C, pH = 7.4, 10 mM phosphate buffer. The concentration of ligand was varied from 1.2 to 75 μM. Inset: Results of fitting the titration data of EPO-P2 antisense with its EPO-P2 ligand at 310 nm, according to the model proposed in Equations (1) and (2).

The binding of peptides was evaluated by means of two methods—tryptophan fluorescence spectroscopy and microscale thermophoresis. Tryptophan fluorescence data were characterized with the SPECFIT software [4,26,27,28,29]. Singular value decomposition analysis suggested only two spectrally active species, one of the antisense peptide DFDIWPLRTAWPLS and the other of its complex with EPO-P2 binding partner, since EPO-P2 is not spectrally active in fluorescence mode. The results, presented in Figure 5, suggested 1 to 1 complex formation, without any higher order complexes. This model is given by Equations (1) and (2), where *K*_d_ is the dissociation constant of the complex:

ANTISENSE − EPO-P2 *⇄* ANTISENSE + EPO-P2 (1)

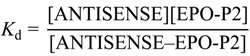
(2)

Calculated dissociation constant (*K*_d_) was 850 ± 160 µM (mean ± SD).

**Figure 6:**
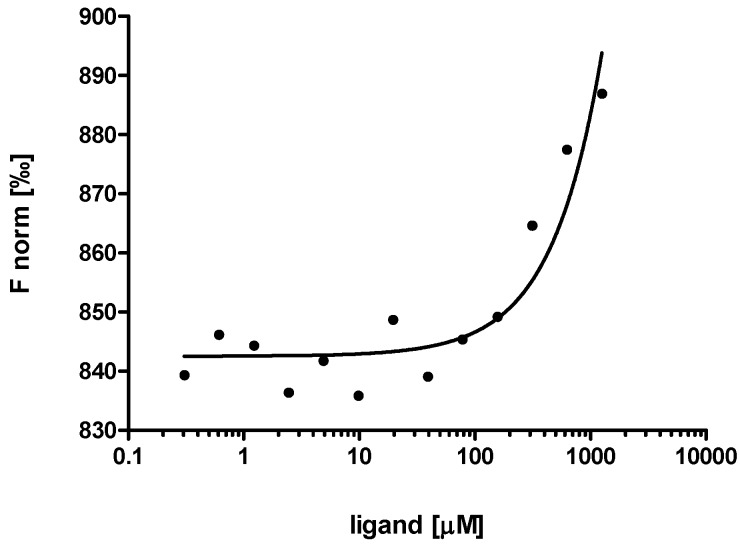
Microscale thermophoresis method confirmed the results of fluorescence spectroscopy titration presented in Figure 5, thus confirmed the binding of antisense paratope DFDIWPLRTAWPLS and its complementary epitope LKLYTGEACRTGDR.

Microscale thermophoresis [30,31,32] was also used to analyze the binding of EPO-P2 antisense peptide DFDIWPLRTAWPLS to EPO-P2 epitope LKLYTGEACRTGDR [30,31,32]. The data, presented in Figure 6, show a single binding event in a micromolar concentration range of the titrant. The dissociation constant of the complex (*K*_d_ = 816 ± 32 µM) was similar to that obtained by means of the tryptophan fluorescence spectroscopy, presented in Figure 5.

Different methods have been used to evaluate sense–antisense peptide interactions, like microtiter plate assay method (immunoassay) and high-performance affinity chromatography, and related techniques [20,33,34,35]. Useful spectroscopical methods for this type of analysis are biosensor based surface plasmon resonance and resonant mirror analyses, electrospray ionization mass spectrometry, and NMR spectroscopy [1,36,37,38,39,40]. Classical biochemical methods such as enzyme linked immunosorbent assay (ELISA) and electrophoretic mobility shift assay (EMSA) are not always repeatable or generalisable when complementary peptide binding is observed [7,41]. Similar is valid for the chromatographic techniques [7].

Tryptophan fluorescence method and microscale thermophoresis that we used in this study proved to be simple, efficient and sensitive. Their advantage is that the measurement is done within the range of physicochemical parameters that resemble physiological conditions [4], and the volume and quantity of the sample is small, especially for microscale thermophoresis [30,31,32]. This fact is important in the case of screening a large number of compounds. This represents an advance over the common nonfuorescent physicochemical methods such as isothermal titration calorimetry (ITC) or surface plasmon resonance (SPR) [41,42]. Tryptophan fluorescence titration and microscale thermophoresis can be performed in different buffers, body fluids and cell suspensions/lysates [4,30,31,32,41,42,43,44,45]. Therefore, they provide a novel, immobilization-free and label-free physicochemical approach to study molecular interactions of small peptides [41,42].

Fluorescence as a method depends only on the change in the fluorophore environment and not on the size or type of the molecules [45]. Consequently, it is suitable for the detection of protein–protein and peptide–peptide interactions, as well as the quantification of these affinities (*K*_d_) [4,45]. Microscale thermophoresis also turned out to be a very appropriate method for the evaluation of peptide–peptide interactions, due to large change in the relative size/diffusion of the molecules as reactants and as a product species [41,42,43].

## 3. Experimental Section

### 3.1. Erythropoietin (EPO) Molecule Structure

Primary, secondary and tertiary structures of the EPO molecule (PDB file 1BUY) are presented in Figure 1 using Unipro UGENE software [46,47]. Five principal epitopes of the EPO (P1–P5) were predicted with the computer program of the University of Wisconsin Genetics Computer Group [11,14]. UWGCG software package is a classic collection of algorithms for protein structure/function studies including the plots of: hydrophilicity according to Hopp and Woods (Table 1), surface probability (Emini *et al.*), flexibility (Karplus and Schulz), antigenic index (Jameson and Wolf) and secondary structure (CF, GOR) [12,14]. In this type of analysis basic information obtained from the molecular hydrophilicity is supplemented by other parameters [12,14].

### 3.2. Resonant Recognition Method (RRM)

Primary amino acid sequence of the EPO molecule was converted into the numerical series using Resonant Recognition Method (RRM) [15]. EIIP value was assigned to each amino acid [15]. The values of EEIP for 20 amino acids and EPO sequence are given in Table 1. The informational spectrum (IS) of the protein sequence in Figure 2 was calculated by means of a single-series Fourier analysis in order to obtain highest frequency peaks of the periodogram. According to the theoretical concept of RRM those highest peaks (also named *hot spots*) often belong to the bioactive part of the molecule (Figure 2) [15]. Peak position = 2 × Frequency × sequence length. Software STATISTICA for Windows version 8.0 was used for the analysis [48].

### 3.3. Peptides

EPO peptide of the P2 region aa 152–166 (EPO-P2, Figure 1). Sequence: LKLYTGEACRTGDR (*M*_W_ 1582.65, >97% purity; GenScript, Piscataway, NJ, USA). Antisense peptide of the EPO-P2 region. Sequence: DFDIWPLRTAWPLS (*M*_W_ 1716.50, >97% purity; GenScript).

### 3.4. Circular Dichroism Spectroscopy

The circular dichroism spectra were measured with Jasco J-815 CD spectrometer equipped with thermostated cell holder in a rectangular 1 cm cuvette (Jasco Inc., Easton, MD, USA). Conditions: concentration of peptides = 1 mg/mL, phosphate buffer = 0.1 M, pH = 7.4, temperature = 25 °C. Spectra are presented as averages of five scans, and corrected for buffer spectrum.

### 3.5. Tryptophan Fluorescence Spectroscopy

Fluorescence spectra of the binding peptides and their complexes were measured at 25 °C by OLIS RSM 1000F spectrofluorimeter (Olis, Inc., Bogart, GA, USA) equipped with thermostatted cell holder. The excitation wavelength was 280 nm. Antisense peptide to EPO-P2 and its complex exhibited fluorescence, whereas the EPO-P2 ligand did not. Fluorescence units are given as a ratio of signals obtained from sample and reference PMTs. Data obtained from the titrations were analyzed with SPECFIT software [4,26,27,28,29].

### 3.6. Microscale Thermophoresis

Microscale thermophoresis experiment of the peptide binding was performed with use of the Monolith.NT.LabelFree instrument by measuring gradual thermophoretic pattern changes through detection of intrinsic tryptophan fluorescence (NanoTemper Technologies GmbH, Munich, Germany) [30,31,32], in context of EPO-P2 titration into reaction mixture. The concentration of EPO-P2 antisense peptide DFDIWPLRTAWPLS containing tryptophan was kept constant (10 µM), while the concentration of its binding partner, *i.e.*, non-fluorescent EPO-P2 peptide was varied between 10 and 0.305 µM. A serial dilution of the EPO-P2 peptide (titrant) was prepared starting from 10 mM in phosphate buffer. After a short incubation (15 min) the samples were loaded into microscale thermophoresis NT.LabelFree standard glass capillaries, and the experiment was performed.

## 4. Conclusions

We present a simple and efficient three step procedure for the antisense peptide design and the verification of its binding using the model of human EPO molecule.

(1) In the first step the receptor binding region of the EPO molecule was selected as a template for the antisense peptide modeling.

(2) The second step was the rational design of the antisense peptide DFDIWPLRTAWPLS (paratope) directed to the selected EPO region (epitope). The method combined antisense mRNA transcription of the epitope template in the 3'→5' direction, and computational screening of potential paratope structures using Basic Local Alignment Search Tool (BLAST).

(3) The third step was the evaluation of sense–antisense (epitope–paratope) peptide binding by means of the fluorescence spectroscopy and microscale thermophoresis. The advantages of the methods were: fast screening with a small quantity of the sample needed, and measurements done within the range of physicochemical parameters resembling physiological conditions.

A simple three-step method presented in this study enables fast, inexpensive and reliable selection of antisense peptides with high affinity for specific targets. Antisense peptides to specific EPO region(s) could be used for the development of new immunochemical methods that avoid binding problems caused by glycosylation or sequence modification.

The current accent in pharmacological research is modulation of protein–protein interactions, and the use of complementary peptides provides insight in such systems [49,50]. The method may be easily adapted for high-throughput screening, especially in the context of microscale thermophoresis equipment developed for this purpose [42]. Selected antisense peptides with optimal affinity could be used as a starting point for the development of novel assays, vaccines, biopharmaceuticals and diagnostic substances. However, additional investigations of selectivity and pharmacokinetic properties will be required for specific purposes.
